# Unveiling the role of Jagged2 in hypoxic pulmonary arterial hypertension: A NOX2‐mediated pathway

**DOI:** 10.1002/ccs3.70032

**Published:** 2025-11-19

**Authors:** Jieqing Yuan, Yunfeng Chen, Siyan Wu, Hai Shi, Yuan Dong, Yu Han, Wenjie Cui

**Affiliations:** ^1^ Department of Respiratory and Critical Care Medicine The Affiliated Xuzhou Municipal Hospital of Xuzhou Medical University Xuzhou China; ^2^ Department of Respiratory and Critical Care Medicine Xuzhou First People's Hospital Xuzhou China

**Keywords:** Jagged2, NADPH oxidase 2, pulmonary arterial hypertension, reactive oxygen species, vascular inflammation

## Abstract

Hypoxic pulmonary arterial hypertension (PAH) is a severe cardiovascular condition involving vascular remodeling and inflammation. Jagged2 (Jag2) has been implicated in various pathologies but its role in PAH remains unclear. We integrated bioinformatics analysis of transcriptomic data with in vitro and in vivo experiments to investigate Jag2's function in hypoxic PAH. We focused on primary rat pulmonary artery smooth muscle cells (PASMCs) for cellular responses and a rat model for hemodynamic changes. Jag2 was upregulated under hypoxic conditions, promoting PASMC proliferation and migration and inhibiting apoptosis through NADPH oxidase 2 (NOX2)/reactive oxygen species (ROS) signaling. Inhibition of Jag2 ameliorated hemodynamic changes and vascular remodeling in the PAH rat model. Jag2 activation of NOX2/ROS signaling is a critical driver of vascular inflammation and remodeling in hypoxic PAH, suggesting the Jag2/NOX2 axis as a therapeutic target.

## Graphical abstract

**Molecular Mechanism of Jag2 Activation of the NOX2/ROS Pathway in Hypoxic PAH Pathology**. This study provides important insights into the mechanism of Jag2 in PAH, particularly its critical role in vascular inflammation and remodeling through the NOX2/ROS pathway. This discovery offers a new direction for PAH pathology research and establishes a theoretical basis for developing Jag2‐targeted therapies. Inhibiting Jag2 may effectively mitigate PAH progression and improve patient outcomes. However, the main limitation of this study lies in the experimental models and cell lines used. The results are primarily based on rat models and in vitro cell experiments, which may differ from human physiological and pathological conditions. Although our data strongly support the role of the Jag2/NOX2/ROS pathway in PAH, further studies are needed to validate its generality and specific mechanisms across different PAH subtypes. Future research should focus on clinical validation, evaluating the safety and efficacy of Jag2 inhibitors in PAH patients and exploring other molecular mechanisms that may contribute to PAH pathology for a comprehensive understanding and treatment of PAH.

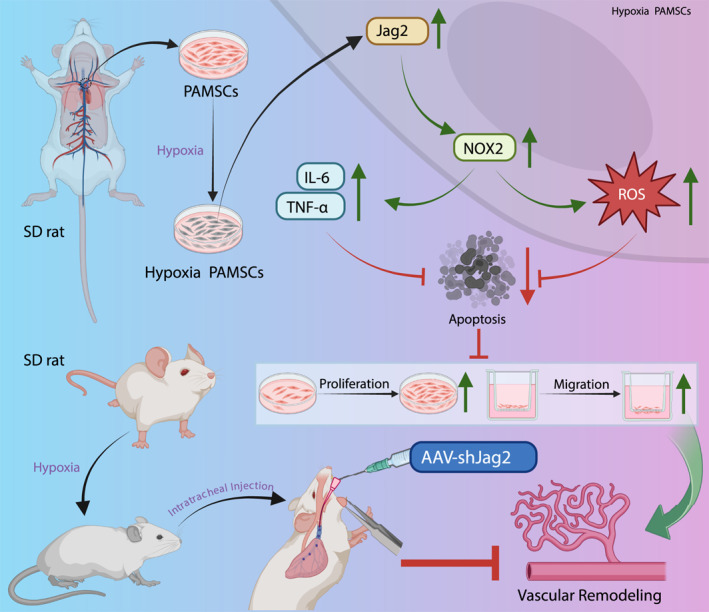

## 1 INTRODUCTION

Pulmonary arterial hypertension (PAH) is a severe cardiopulmonary disease characterized by elevated pulmonary artery pressure. Its pathophysiological features include increased pulmonary vascular resistance, right ventricular hypertrophy, and cardiac dysfunction.^1^, ^2^, ^3^ The global incidence of PAH is steadily increasing, posing a substantial threat to patients' quality of life and life expectancy.^1^, ^4^, ^5^ Current statistics indicate that the 5‐year survival rate for individuals with PAH remains below 50%, highlighting the severe impact of the disease on patients’ well‐being and underscoring its considerable socioeconomic burden.^4^, ^6^, ^7^ The etiology of PAH is complex and multifactorial, involving genetic, environmental, immunological, and molecular factors that contribute to its onset and progression.^1^, ^4^, ^8^ Studies indicate that endothelial dysfunction, smooth muscle cell proliferation and migration, inflammatory responses, and oxidative stress play crucial roles in the pathogenesis of PAH.^9^, ^10^, ^11^ However, the precise mechanisms underlying PAH remain incompletely understood and require further investigation.

Currently, the primary treatments for PAH include medication, surgical interventions, and lung transplantation.^12^, ^13^, ^14^ However, these treatments have significant limitations; they do not effectively reverse the disease progression and only alleviate symptoms and extend the patient's lifespan.^12^, ^13^, ^14^ For severe PAH patients, the efficacy of existing treatments is often limited, highlighting the urgent need for new therapeutic strategies.^15^, ^16^ Recently, targeted therapies focusing on molecular and signaling pathways have become a research hotspot in PAH.^17^, ^18^, ^19^ Advances in bioinformatics have made it possible to gain deeper insights into the molecular mechanisms of PAH.^20^, ^21^, ^22^ Bioinformatics can aid in identifying candidate genes involved in the pathogenesis of PAH, offering valuable guidance for the discovery of diagnostic or prognostic biomarkers and potential therapeutic targets.^20^, ^23^ However, many current studies focus solely on predictive analysis without experimental validation to confirm the reliability of their findings.^22^, ^24^

In this study, we applied bioinformatics analysis to explore the molecular mechanisms associated with PAH under hypoxic conditions. Two transcriptomic microarray datasets (GSE72181 and GSE186996) were retrieved from the Gene Expression Omnibus (GEO) database, and differential expression analysis was performed to identify significant differentially expressed genes (DEGs) between the hypoxia and normoxia groups. Kyoto encyclopedia of genes and genomes (KEGG) and gene ontology (GO) enrichment analyses were subsequently conducted to interpret the biological processes (BP) and pathways involved. Furthermore, we constructed a weighted gene co‐expression network (WGCNA) to identify gene modules highly correlated with hypoxia. Key genes were evaluated and selected using machine learning algorithms including random forest (RF) and least absolute shrinkage and selection operator regression (LASSO). The findings from the bioinformatics analysis were validated through both in vitro and in vivo experiments, systematically assessing the role of the Jag2‐NOX2 axis in hypoxia‐induced pulmonary artery smooth muscle cell (PASMC) proliferation, migration, apoptosis, oxidative stress, and vascular remodeling.

This study aims to elucidate the pathogenic mechanisms of PAH and identify potential therapeutic targets, thereby providing a scientific foundation for developing novel molecular‐targeted therapies.

## 2 MATERIALS AND METHODS

### 2.1 Public data download

Transcriptome expression data for hypoxic PAH were downloaded from the GEO database (https://www.ncbi.nlm.nih.gov/geo/). Multiple datasets related to hypoxic PAH were selected, including GSE72181^25^ and GSE186996,^26^ with sample information provided in Table S1. These datasets included samples from both hypoxic PAH and normoxic control groups. The selected datasets were comprehensive, with high sequencing depth, and provided robust molecular‐level evidence to support the study. The ComBat algorithm from the “sva” R package (v3.5.0.0) was employed to merge the two datasets into a new matrix to eliminate batch effects.

DEGs were identified using the “limma” R package (v3.58.1) with a threshold of 1.5‐fold change and *p* < 0.05.

### 2.2 GO and KEGG enrichment analysis

Gene set functional enrichment analysis was performed using the R package org.Hs.eg.db (version 3.1.0) for GO annotations, which were utilized as the background for mapping the genes into the background set. Enrichment analysis was then conducted using the R package clusterProfiler (version 3.14.3) to obtain results of gene set enrichment. Additionally, the latest KEGG pathway gene annotations were accessed via the KEGG REST API for the enrichment analysis. These annotations served as the background, and clusterProfiler (version 3.14.3) was again employed for the enrichment analysis. The minimum gene set size was set to 5 and the maximum to 5000, with a *p*‐value threshold of <0.05 and a false discovery rate of <0.25. Data visualization was conducted using the ggplot2 package.

### 2.3 WGCNA

Gene expression profiles were utilized to calculate the median absolute deviation (MAD) for each gene. The 50% of genes with the smallest MAD values were excluded, and the goodSamplesGenes function in the WGCNA R package was used to remove outlier genes and samples. Subsequently, WGCNA was employed to construct a scale‐free co‐expression network, with a minimum module size of 30 and a sensitivity of 3. Modules with a distance of less than 0.25 were merged, resulting in 12 co‐expression modules. The gray module was identified as a collection of genes that could not be assigned to any other module.

Pearson correlation tests (*p* < 0.05) were performed to analyze the relationship between modules and groups. The genes in the most significant module were identified as hypoxia‐related genes for further analysis.

The VennDiagram package (v1.7.3) in R was used to obtain the intersection of significant WGCNA module genes and DEGs.

### 2.4 Machine learning for identifying key factors

LASSO regression, a method used for variable selection and regularization in regression models, was employed. Cross‐validation was used to select the optimal regularization parameter (*λ*), fitting the model across a range of *λ* values and selecting the one that performed best. Coefficients were examined at the chosen *λ* value. LASSO regression typically shrinks some coefficients to zero, facilitating variable selection. The glmnet R package was used to perform LASSO regression analysis.

The RF algorithm, a machine learning method, was applied for key factor screening within the R environment. This algorithm analyzed transcriptomic data to identify critical factors in the hypoxia group. RF integrates multiple decision trees to form a forest, predicting the final outcome. The randomForest package was used to conduct RF analysis.

### 2.5 Receiver operating characteristic (ROC) curve analysis and survival analysis

The accuracy of disease status prediction based on candidate gene expression levels was evaluated using both training and validation datasets. The performance of gene expression in distinguishing between different disease states was assessed by plotting ROC curves using the pROC package in R.

### 2.6 Isolation and culture of PASMCs

Sprague Dawley (SD) rats (6–8 weeks old, 200–250 g) were purchased from Vital River Laboratories for the isolation of PASMCs. The rats were disinfected with 75% ethanol and dissected under sterile conditions. Pulmonary arteries were excised from the distal lung lobes, and surrounding fat and connective tissues were removed. The arteries were transferred into phosphate‐buffered saline (PBS) containing 1% penicillin‐streptomycin for 5 min, rinsed with cold PBS three times, and the intima and adventitia were carefully removed under a microscope.^27^ The arteries were cut into ∼2 mm segments and incubated in Hank's balanced salt solution containing type II collagenase (1 mg/mL, 1148090, Sigma) and elastase (1 mg/mL, E7885, Sigma) at 37°C for 1–2 h. PASMCs were collected by centrifugation at 1200 rpm for 5 min and cultured in Dulbecco's modified Eagle medium (DMEM, 11965118, ThermoFisher) supplemented with 15% fetal bovine serum (FBS, A5669701, Gibco). To identify PASMCs, alpha‐smooth muscle actin (α‐SMA) positive staining was performed as follows: The isolated PASMCs were seeded on coverslips and fixed with 4% paraformaldehyde for 20 min. The cells were then permeabilized with Triton X‐100 for 20 min. After blocking with goat serum (C0265, Beyotime Biotechnology) for 30 min, the cells were incubated overnight at 4°C with the primary antibody against α‐SMA (14395‐1‐AP, Proteintech). The following day, the cells were incubated with FITC‐conjugated goat anti‐rabbit IgG secondary antibody (SA00003‐2, Proteintech) for 1 h. Finally, the cells were incubated with DAPI (p0131, Beyotime Biotechnology) for 5 min and observed under a fluorescence microscope (DM1000, Leica). The purity of α‐SMA‐positive cells was ≥99%.

PASMCs were cultured in DMEM containing 15% FBS and 1% penicillin‐streptomycin at 37°C and 95% humidity. PASMCs were subjected to normoxic treatment with 5% oxygen or hypoxic treatment in a gas‐tight chamber (Billups‐Rothberg) containing 5% carbon dioxide and 90% nitrogen. The cells were divided into the normoxia group and several hypoxia groups: hypoxia (Hypoxia), normoxia with NADPH oxidase 2 (NOX2) overexpression (oe‐NOX2), hypoxia with control siRNA (Hypoxia + siNC), hypoxia with Jag2 knockdown (Hypoxia + siJag2), hypoxia with Jag2 knockdown and control overexpression (Hypoxia + siJag2 + oeNC), and hypoxia with Jag2 knockdown and NOX2 overexpression (Hypoxia + siJag2 + oeNOX2).

### 2.7 Plasmid construction and transfection

The siRNA sequences targeting rat Jag2 and the negative control sequences were transfected using Lipofectamine™ 3000 reagent (L3000001, ThermoFisher). The sequences for siJag2 are as follows: siJag2‐1, TGCAAAGAAGCCGTGTGTAAACA; siJag2‐2, GTGTAAACAAGGATGTAATTTGC; siJag2‐3, GGCAAGTTCTGTGATGAATGTGT. The negative control sequence is TTCTCCGAACGTGTCACGT. The silencing efficiency was validated by the western blot and reverse transcription quantitative polymerase chain reaction (RT‐PCR). The cDNA fragment of NOX2 (NCBI reference sequence: NM_023965) was inserted into the pcDNA3.1 vector and transfected using Lipofectamine™ 3000 reagent.

### 2.8 CCK‐8 cell viability assay

PASMCs were seeded into 96‐well plates at a density of 3 × 10^3^ cells per well. At the designated time points, the culture medium was removed and replaced with 100 μL of fresh medium containing 10 μL of CCK‐8 solution (HY‐K0301, MCE). The cells were incubated at 37°C for 1.5 h. Absorbance was then measured at 450 nm using a BioTek microplate reader.

### 2.9 5‐Ethynyl‐2′‐deoxyuridine (EdU) staining

After PASMCs infected with the corresponding siRNA were treated under normoxic or hypoxic conditions, the cells were incubated with 50 μM EdU (C00003, RiboBio) for 2 h. The cells were then fixed with 4% paraformaldehyde and incubated in the dark with 100 μL of 1 × Apollo® solution for 30 min. The cell nuclei were stained by adding 100 μL of 1 × Hoechst 33342 to each well. The cells were observed using a fluorescence microscope (Olympus).

### 2.10 Cell migration assay

Cells from each group were seeded into the upper chambers of 24‐well Transwell inserts. The lower chambers were filled with 600 μL of DMEM. After 24 h, the medium in the upper chambers was replaced with serum‐free medium, whereas the lower chambers were refilled with medium containing 15% FBS for an additional 12 h. Migrated cells were fixed with 4% paraformaldehyde and stained with 10% crystal violet (HY‐B0324A, MCE) for 15 min. The cells were then counted under an optical microscope (IX71, Olympus).

### 2.11 Detection of cell apoptosis by flow cytometry (FCM)

After treating PASMCs infected with specific siRNAs under normoxic or hypoxic conditions, both floating and adherent cells were collected. These cells were incubated with 5 μL of Annexin V‐FITC and 5 μL of PI (HY‐K1073, MCE) in the dark for 15 min. Apoptotic cells were then detected using a flow cytometer (BD Biosciences), and the percentages of early apoptotic (Annexin V+/PI−) and late apoptotic (Annexin V+/PI+) cells were analyzed.

### 2.12 TUNEL assay for detecting cell apoptosis

After PASMCs infected with the corresponding siRNA were treated under normoxic or hypoxic conditions, the cells were incubated with the terminal deoxynucleotidyl transferase dUTP nick end labeling (TUNEL) assay kit (HY‐K1079, MCE) for 1 h in a dark environment. The cell nuclei were then stained with DAPI (p0131, Beyotime). The fluorescence was observed under a fluorescence microscope (Olympus).

### 2.13 Measurement of cellular reactive oxygen species (ROS) levels

After PASMCs infected with the corresponding siRNA were treated under normoxic or hypoxic conditions, the cells were incubated with 10 μM 2',7'‐dichlorodihydrofluorescein diacetate (DCFH‐DA) solution (D6883, Sigma) for 20 min. The cells were then washed three times with DMEM without FBS. Fluorescence was observed under a fluorescence microscope (Olympus), and the fluorescence intensity was quantified using Image‐Pro Plus 6.0.

### 2.14 Animal housing and grouping

Thirty‐two male Sprague Dawley (SD) rats (6–8 weeks old, 200–250 g) were purchased from the Experimental Animal Center of Tongji Medical College. The rats were randomly divided into four groups, with eight rats per group: control (normoxia), model (hypoxia), model + adeno‐associated virus (AAV)‐shNC, and model + AAV‐shJag2. Before establishing the hypoxia‐induced PAH model,^28^, ^29^ the AAV‐shNC and AAV‐shJag2 groups received an intratracheal injection of 25 μL 1.75E+13 v.g/mL AAV virus (AAV1 virus purchased from Hanbio Biotechnology). The AAV‐shNC rats were injected with a negative control shRNA (sequence: TAAGGCTATGAAGAGATAC), whereas the AAV‐shJag2 rats received a shRNA targeting Jag2 (sequence: GCGTGGACCTTCACCTTCAAA). The control group was maintained under normoxic conditions (21% oxygen), whereas the model, model + AAV‐shNC, and model + AAV‐shJag2 groups were kept in a hypoxia chamber (Oxycycler model A84XOV; BioSpherix, Lacona, NY) with an oxygen concentration of 10% ± 0.5%. Calcium hydroxide was placed in the chamber to absorb carbon dioxide, keeping its concentration below 3%, and color‐changing silica gel was used to absorb water vapor. After 4 weeks of hypoxia treatment, the rats were euthanized, and their tissues and organs were collected for subsequent experiments.^30^, ^31^

### 2.15 Hemodynamic parameter measurement

After 4 weeks of hypoxia treatment, the rats in each group (for specific grouping, see “Animal Breeding and Grouping” section) were anesthetized with an intraperitoneal injection of sodium pentobarbital (40 mg/kg) (P3761, Sigma). A heparin‐filled venous catheter (PE‐50, Taimeng, Chengdu) was inserted via the right jugular vein and positioned appropriately. The other end of the catheter was connected to a pressure transducer linked to a multichannel physiological recorder (BL‐420S, Taimeng, Chengdu) to measure and record the mean pulmonary arterial pressure (mPAP), right ventricular systolic pressure (RVSP), and pulmonary artery systolic pressure (PASP). Subsequently, the heart and lung tissues were collected, and the right ventricle (RV) was carefully separated from the heart. The RV, left ventricle (LV), and septum (S) were weighed. The degree of right ventricular hypertrophy was determined by the ratio of the RV mass to the combined mass of the LV and S (RV/[LV + S]).

### 2.16 Morphological measurements

After fixing the lung tissues of each group of rats with 4% paraformaldehyde (P0099, Beyotime Biotechnology), the tissues were dehydrated through an ethanol gradient and embedded in paraffin. The lung tissues were then sectioned into 5 μm slices. These sections were stained using Elastica Van Gieson (EVG) stain (DC0049, Reagan Biotechnology) and H&E stain (C0105S, Beyotime Biotechnology). Observations were made using an optical microscope (Leica 3000B). H&E staining results allowed for the measurement of the medial arterial wall thickness to medial arterial diameter ratio (WT%) and the medial arterial wall area to medial arterial area ratio (WA%). EVG staining was used to observe the medial area and total vascular area of the pulmonary arterioles, which were evaluated for vascular remodeling.

### 2.17 Immunofluorescence staining

Lung tissues from each group of rats were fixed in 4% paraformaldehyde, embedded in OCT compound, and cut into 7 μm thick frozen sections. The sections were permeabilized with 0.25% Triton X‐100 (P0096, Beyotime Biotechnology) and blocked with 5% BSA (ST023, Beyotime Biotechnology). The sections were then incubated overnight at 4°C with rabbit anti‐CD31 (28083‐1‐AP, Proteintech) and mouse anti‐α‐SMA (67735‐1‐lg, Proteintech) primary antibodies. Subsequently, the sections were incubated for 2 h with FITC‐conjugated goat anti‐rabbit IgG secondary antibody (SA00003‐2, Proteintech) and Coralite 594‐conjugated goat anti‐mouse IgG secondary antibody (RGAM004, Proteintech). The nuclei were stained with DAPI (p0131, Beyotime Biotechnology). The cell morphology was observed under a fluorescence microscope, and the fluorescence intensity was quantified using Image‐Pro Plus 6.0 software.

### 2.18 Immunohistochemistry staining

Lung tissues from each group of rats were fixed in 4% paraformaldehyde, dehydrated through an ethanol gradient, and embedded in paraffin to create tissue sections. The lung tissues were sliced into 5 μm sections and incubated with 3% hydrogen peroxide. Antigen retrieval was performed using sodium citrate (P0081, Beyotime Biotechnology). The sections were then blocked with 5% BSA and incubated overnight at 4°C with the primary antibody CD68 (28058‐1‐AP, Proteintech). The next day, the sections were incubated with the secondary antibody (RGAR011, Proteintech) at room temperature, followed by DAB staining (P0203, Beyotime Biotechnology) and hematoxylin counterstaining. Observations were made using an optical microscope (IX71, Olympus), and fluorescence intensity was quantified using Image‐Pro Plus 6.0.

### 2.19 Transmission electron microscopy

To observe the morphology of the basal membrane in rat pulmonary arterioles, lung tissues from each group were placed in 0.1M PBS buffer (pH 7.2). The tissues were then trimmed and fixed in precooled 3% glutaraldehyde (G5882, Sigma). Subsequently, the sections were stained with 2% uranyl acetate (U25690, ACMEC) and 0.2% lead citrate (17800, Electron Microscopy Sciences) and examined using a transmission electron microscope (Zeiss Libra).

### 2.20 Measurement of oxidative stress markers

Following the kit manufacturer's instructions, the levels of superoxide dismutase (SOD, A001‐3‐2, NJJCBIO), malondialdehyde (MDA, A003‐1‐2, NJJCBIO), total glutathione (GSH, A061‐1‐1, NJJCBIO), and reduced GSH (GSH, A006‐2‐1, NJJCBIO) were measured in serum and lung tissue samples. Optical density (OD) values were determined using a spectrophotometer (Perkin Elmer).

### 2.21 Enzyme‐linked immunosorbent assay (ELISA) detection

Following the manufacturer's instructions, we used ELISA kits to measure the levels of Interleukin‐6 (IL‐6) (BMS625, Invitrogen) and tumor necrosis factor‐alpha (TNF‐α) (BMS622) in serum and lung tissue samples. The optical density (OD) values were measured using a spectrophotometer (Perkin Elmer).

### 2.22 Detection of ROS in pulmonary arteries

The isolated pulmonary arteries were placed in 10 μM DHE dye (HY‐D0079, MCE) and subjected to normoxic or hypoxic conditions for 1 h. Excess dye was washed away using a Krebs‐Ringer bicarbonate solution. The arteries were then sectioned into 7 μm frozen slices and observed under a confocal microscope (Leica Microsystems). Changes in ROS levels were indicated by the ratio of EtBr to DHE fluorescence intensity.

### 2.23 TUNEL assay for pulmonary artery apoptosis

Lung tissues from each group of rats were prepared into 7 μm consecutive sections and permeabilized with 0.25% Triton‐X 100. After blocking with 5% BSA, the sections were incubated overnight with the primary antibody mouse anti‐α‐SMA (67735‐1‐lg, Proteintech). The following day, the sections were incubated for 2 h with the secondary antibody goat anti‐mouse IgG conjugated with Coralite 594 (RGAM004, Proteintech). Subsequently, the sections were incubated for 1 h at 37°C in the dark with a nucleotide mixture labeled with TdT (HY‐K1078, MCE). Finally, the cell nuclei were stained with DAPI (p0131, Beyotime) and observed under a confocal microscope (Zeiss LSM 880). The fluorescence intensity was quantified using Image‐Pro Plus 6.0.

### 2.24 Reverse transcription quantitative polymerase chain reaction (RT‐qPCR)

Total RNA was extracted from cells using TRIzol reagent (15596026, Invitrogen), and the concentration and purity of the RNA were assessed using a Nanodrop 2000 spectrophotometer (1011U, Nanodrop). The mRNA was reverse‐transcribed into cDNA according to the instructions of the PrimeScript RT Reagent Kit (RR047A, Takara). RT‐qPCR was performed using an ABI7500 real‐time PCR system (7500, ABI) under the following conditions: 95°C for 10 min (initial denaturation), followed by 40 cycles of 95°C for 10 s (denaturation), 60°C for 20 s (annealing), and 72°C for 34 s (extension). Glyceraldehyde‐3‐phosphate dehydrogenase was used as an internal control. Relative gene expression levels were calculated using the 2^−ΔΔCt^ method, where ΔΔCt = ΔCt (experimental group) − ΔCt (control group) and ΔCt = Ct (target gene) − Ct (internal control). Each experiment was repeated three times, and all primers were synthesized by Takara (Table S2).

### 2.25 Western blot analysis

Total proteins were extracted from cells using radioimmunoprecipitation assay buffer lysis buffer containing phenylmethylsulfonyl fluoride (P0013B, Beyotime). The protein concentration was quantified using the bicinchoninic acid assay protein assay kit (23225, Thermo Fisher Scientific). A total of 50 μg of protein was mixed with 2× sodium dodecyl sulfate (SDS) loading buffer and boiled at 100°C for 5 min. The samples were then subjected to SDS‐polyacrylamide gel electrophoresis and transferred onto a polyvinylidene fluoride (PVDF) membrane using the wet transfer method. The membrane was blocked with 5% nonfat dry milk at room temperature for 1 h. The PVDF membrane was incubated overnight at 4°C with primary antibodies diluted in tris‐buffered saline with tween 20 (TBST): Jag2 (bs‐4244R, Bioss), NOX2 (19013‐1‐AP, Proteintech), nuclear factor erythroid 2‐related factor 2 (Nrf2) (16396‐1‐AP, Proteintech), proliferating cell nuclear antigen (PCNA) (10205‐2‐AP, Proteintech), survivin (10508‐1‐AP, Proteintech), SOD2 (24127‐1‐AP, Proteintech), cleaved caspase‐3 (68773‐1‐lg, Proteintech), B‐cell lymphoma 2 (68103‐1‐lg, Proteintech), Bcl‐2‐associated X protein (BAX) (50599‐2‐lg, Proteintech), α‐SMA (14395‐1‐AP, Proteintech), vimentin (10366‐1‐AP, Proteintech), CD31 (28083‐1‐AP, Proteintech), VE‐cadherin (A25003, Abclonal), and β‐actin (81115‐1‐RR, Proteintech). Following primary antibody incubation, the membrane was washed three times with TBST, each wash lasting 10 min. The membrane was then incubated for 1 h at room temperature with an HRP‐conjugated secondary antibody (goat anti‐rabbit IgG H&L, HRP; ab97051, 1:2000, Abcam). After additional washes with TBST, the membrane was prepared for imaging. Equal parts of solutions A and B from the Pierce™ ECL Detection Kit (32209, Thermo) were mixed and applied to the membrane in the dark. Protein bands were visualized using the Bio‐Rad ChemiDoc™ XRS + imaging system (BIO‐RAD).

### 2.26 Statistical analysis methods

Data were obtained from at least three independent experiments, and results are presented as mean ± standard deviation (Mean ± SD). For comparisons between the two groups, an independent samples *t*‐test was used. For comparisons among three or more groups, a one‐way analysis of variance (ANOVA) was performed. If ANOVA indicated significant differences, Tukey's honestly significant difference post hoc test was conducted to compare differences between groups. For data that were not normally distributed or had unequal variances, the Mann–Whitney *U* test or Kruskal–Wallis H test was used. All statistical analyses were performed using GraphPad Prism 9 (GraphPad Software, Inc.) and R software. A significance level of 0.05 was set for all tests, with *p*‐values less than 0.05 considered statistically significant.

## 3 RESULTS

### 3.1 Association of hypoxia‐induced PAH genes with immune response

Two hypoxia‐induced PAH transcriptome datasets, GSE72181 and GSE186996, were successfully obtained from the GEO database, including seven hypoxia samples and seven normoxia samples. All samples were derived from rat lung tissues. The hypoxic samples were established using either chronic hypoxia (10% O_2_ for 4 weeks) or Sugen5416 combined with hypoxia treatment (10% O_2_ for 3 weeks), whereas normoxic samples served as controls without any hypoxic exposure. The “sva” R package was employed to merge these two datasets into a new matrix to eliminate batch effects (Figure S1A,B), resulting in a dataset comprising 14,737 genes (Figure S1C).

**FIGURE 1:**
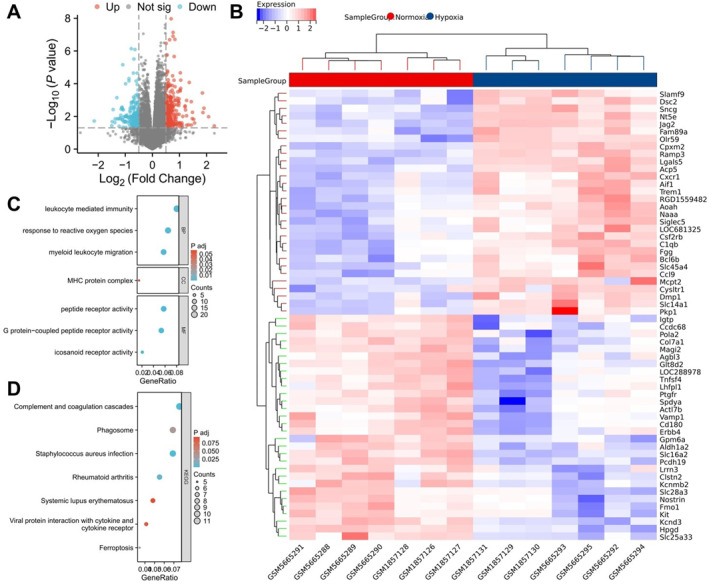
Transcriptomic data analysis revealing molecular mechanisms of hypoxic pulmonary arterial hypertension (PAH). (A) Volcano plot of differential transcriptomic analysis: red indicates significantly upregulated genes, blue indicates significantly downregulated genes, and gray indicates genes with no significant difference; (B) heatmap of the top 30 DEGs; (C, D) Kyoto encyclopedia of genes and genomes (KEGG) (C) and gene ontology (GO) (D) enrichment analysis of differentially expressed genes (DEGs). Normoxia: *n* = 7; Hypoxia: *n* = 7.

Differential expression analysis revealed that, compared to the normoxia group, the hypoxia group had 288 DEGs, with 166 genes upregulated and 122 genes downregulated (Figure 1A,B). Enrichment analysis of these DEGs indicated that they are significantly associated with pathways such as complement and coagulation cascades, viral protein interaction with cytokine and cytokine receptors, and ferroptosis (Figure 1C). Further GO enrichment analysis revealed that, in the BP category, DEGs were significantly associated with leukocyte‐mediated immunity, response to ROS, and myeloid leukocyte migration; in the cellular component category, with the major histocompatibility complex protein complex; and in the molecular function category, with peptide receptor activity, G protein‐coupled peptide receptor activity, and icosanoid receptor activity (Figure 1D).

High‐throughput transcriptome analysis unveiled the gene expression patterns under hypoxic conditions in PAH. The differential expression and subsequent functional enrichment analyses provide new insights into the molecular mechanisms of PAH under hypoxia. Notably, pathways related to immune response, oxidative stress response, and cell death were significantly enriched in the hypoxia group, suggesting that these BP may play crucial roles in the progression of hypoxia‐induced PAH.

### 3.2 Identification of Jag2 as a potential biomarker for hypoxic PAH

**FIGURE 2:**
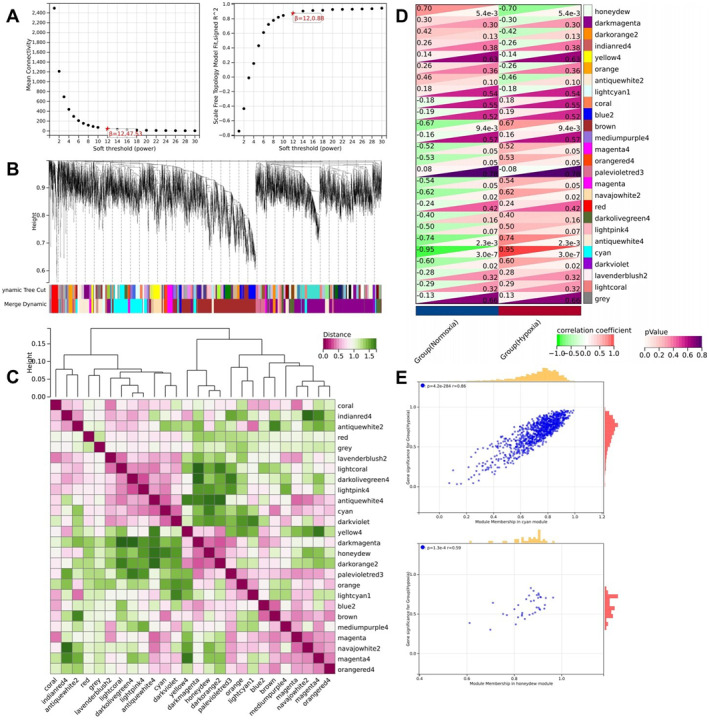
Construction of the co‐expression network identifying key modules in hypoxic pulmonary arterial hypertension (PAH). (A) Scale independence (left) and mean connectivity (right) determined the weighted value *β* = 12 to satisfy the scale‐free network law; (B) dendrogram of co‐expression network modules; (C) correlation heatmap of the 12 modules; (D) correlation analysis of the modules with the hypoxia group; (E) scatter plot analysis of the cyan and honeydew modules with hypoxia. Normoxia: *n* = 7; Hypoxia: *n* = 7.

Using WGCNA, we constructed a comprehensive gene co‐expression network. An optimal soft threshold of *β* = 12 (*R* ^2^ = 0.88) was used to establish a scale‐free network (Figure 2A). Following the merging of modules with highly correlated eigengenes, 12 distinct modules were identified (Figure 2B). Figure 2C illustrates the correlation among these 12 modules utilized for WGCNA analysis.

The module‐trait relationship analysis revealed a significant positive correlation between the cyan module and hypoxia and a significant negative correlation between the honeydew module and hypoxia (Figure 2D,E). This indicates that the 988 genes within these two modules are most likely involved in the development of hypoxic PAH.

**FIGURE 3:**
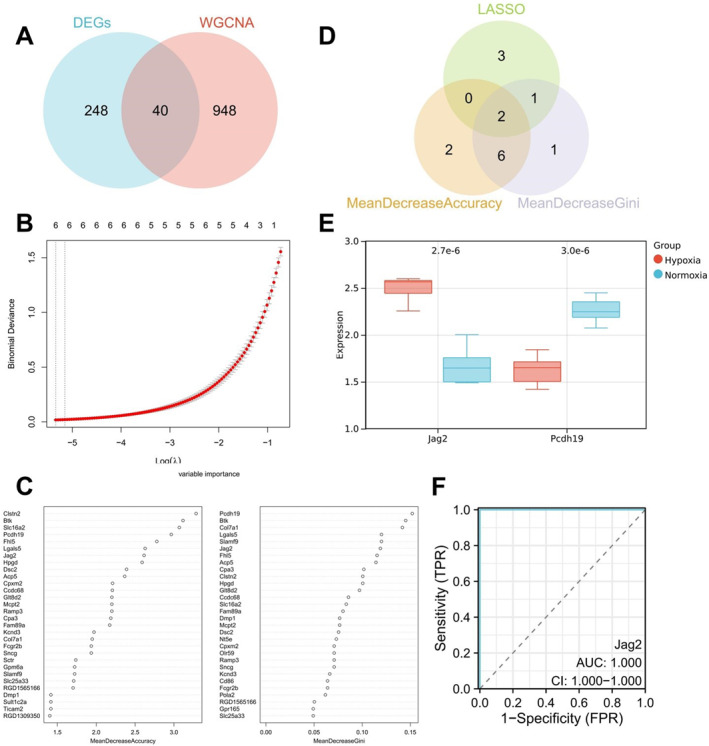
Machine learning identification of key genes. (A) Venn diagram of differentially expressed genes (DEGs) and weighted gene co‐expression network (WGCNA) module genes; (B) least absolute shrinkage and selection operator regression (LASSO) coefficient selection plot; (C) random forest (RF) identification of key factors, with mean decrease accuracy (left) indicating the importance of genes to model prediction accuracy and mean decrease Gini (right) indicating the importance of genes to model classification purity; (D) Venn diagram of LASSO and RF analysis; (E) box plot of Jag2 and Pcdh19 expression in transcriptome datasets; (F) receiver operating characteristic (ROC) curve of Jag2 in transcriptome datasets, with *Y*‐axis representing sensitivity and *X*‐axis representing specificity. Normoxia: *n* = 7; Hypoxia: *n* = 7.

The aforementioned transcriptomic analysis of DEGs and subsequent enrichment analysis indicates that gene regulatory changes under hypoxic conditions involve immune responses. By intersecting DEGs with WGCNA module genes, 40 overlapping genes were identified (Figure 3A). Further, multivariate Cox analysis using LASSO regression on these 40 overlapping DEGs identified six key DEGs (Cpxm2, Jag2, Lgals5, Olr59, Pcdh19, and Slamf9) (Figure 3B).

We further analyzed these 40 genes using the RF machine learning algorithm. By evaluating the importance of features, we found that a higher mean decrease accuracy indicates a greater contribution of the gene to the model's accuracy, and a higher mean decrease Gini signifies a greater contribution to the purity of the classification results (Figure 3C). By intersecting the top 10 genes from both mean decrease accuracy (Clstn2, Btk, Slc16a2, Pcdh19, Fhl5, LgalS5, Jag2, Hpgd, Dsc2, and Acp5) and mean decrease Gini (Pcdh19, Btk, Col7a1, LgalS5, Slamf9, Jag2, Fhl5, Acp5, Clstn2, and Hpgd) with the significant genes identified by LASSO, we ultimately identified two key genes—Jag2 and Pcdh19 (Figure 3D).

Transcriptome analysis revealed that Jag2 was significantly upregulated in the hypoxia group, whereas Pcdh19 was significantly downregulated, with Jag2 showing a more pronounced expression than Pcdh19 (Figure 3E). ROC curve analysis demonstrated that Jag2 had an area under the curve (AUC) value of 1 in distinguishing between normoxia and hypoxia groups, indicating high diagnostic performance (Figure 3F).

Therefore, we identified Jag2 as a key gene in hypoxic PAH. Studies have shown that under hypoxic conditions, Jag2 can influence the downstream Notch signaling pathway, altering the formation of vascular endothelial cells.^32^

By integrating WGCNA and machine learning methods, we identified Jag2 as a potential biomarker for hypoxic PAH, suggesting that it may play a central role in the pathogenesis of PAH under hypoxic conditions through its involvement in the Notch signaling pathway and other cellular processes.

### 3.3 Jag2/NOX2 signaling pathway regulation of PASMCs function under hypoxic conditions

Preliminary bioinformatics data suggest that Jag2 plays a central role in the pathogenesis of PAH under hypoxic conditions. NOX2 is a primary enzyme for ROS production, and our aforementioned enrichment analysis revealed that the DEGs are significantly enriched in ROS‐related BP. To further elucidate the roles of Jag2 and NOX2 in hypoxia‐induced PAH, we isolated primary PASMCs from rats and identified them via α‐SMA staining (Figure S2A). These PASMCs were subjected to hypoxic treatment over various time periods, and the expression levels of Jag2 and NOX2 were measured. The results indicated that both protein and mRNA levels of Jag2 and NOX2 were significantly upregulated in a time‐dependent manner under hypoxic conditions (Figure 5A,B).

**FIGURE 4:**
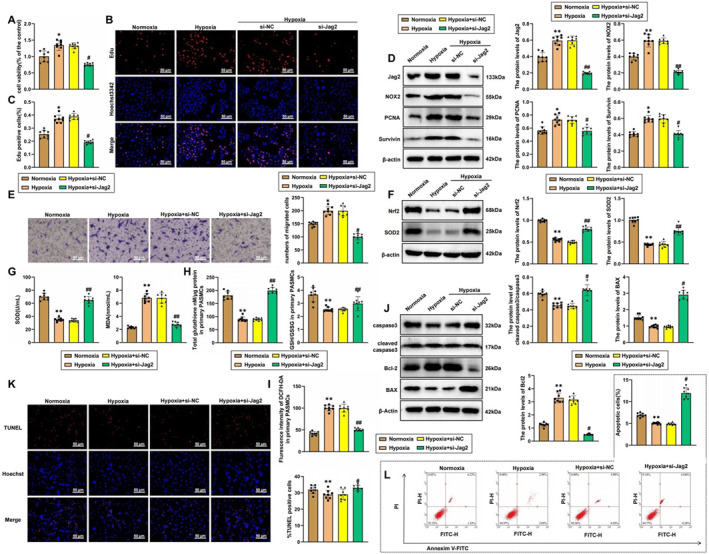
Mechanistic study of Jag2 promoting pulmonary artery smooth muscle cell (PASMC) proliferation and migration under hypoxic conditions through the NADPH oxidase 2 (NOX2)/reactive oxygen species (ROS) pathway. (A) CCK‐8 assay to measure cell proliferation levels in each group; (B, C) EdU staining to measure cell proliferation levels in each group (B), with panel C showing the bar graph statistical results of panel B, scale bar = 50 μm; (D) western blot analysis of Jag2, NOX2, proliferating cell nuclear antigen (PCNA), and survivin protein expression in each group; (E) Transwell assay to measure migration levels in each group; (F) western blot analysis of nuclear factor erythroid 2–related factor 2 (Nrf2) and SOD2 protein expression in each group; (G) kit assays to measure superoxide dismutase (SOD) and malondialdehyde (MDA) levels in each group; (H) kit assays to measure total glutathione (GSH), reduced GSH, and oxidized GSH levels in each group; (I) 2',7'‐dichlorodihydrofluorescein diacetate (DCFH‐DA) staining to measure ROS levels in each group; (J) western blot analysis of caspase‐3, cleaved caspase‐3, B‐cell lymphoma 2 (Bcl2), and Bcl‐2‐associated X protein (BAX) expression in each group; (K) terminal deoxynucleotidyl transferase dUTP nick end labeling (TUNEL) assay to measure apoptosis levels in each group, scale bar = 50 μm; (L) flow cytometry (FCM) to measure apoptosis levels in each group. *p < 0.05 compared to the normoxia group, **p < 0.01 compared to the normoxia group, #*p* < 0.05 compared to the hypoxia + siNC group, ##*p* < 0.01 compared to the hypoxia + siNC group. All experiments were repeated three times.

To further confirm the regulatory role of the Jag2/NOX2 pathway in PASMCs, we constructed Jag2 knockdown PASMCs for subsequent experiments (Figure S2B). Initially, we assessed the impact of Jag2 on PASMC proliferation using CCK‐8 and EdU staining. The CCK‐8 results showed that cell proliferation was significantly enhanced in the hypoxia group compared to the normoxia group, whereas Jag2 knockdown resulted in a marked decrease in PASMC proliferation (Figure 4A). EdU staining revealed a similar pattern, with a substantial increase in EdU‐positive cells under hypoxic conditions, which was significantly reduced upon Jag2 knockdown (Figure 4B,C). Western blot analysis confirmed that hypoxia induced the expression of proliferation markers PCNA and survivin compared to normoxia, but these expressions were notably decreased following Jag2 knockdown. Additionally, NOX2 expression was significantly reduced in Jag2 knockdown cells (Figure 4D). The Transwell assay demonstrated that hypoxia enhanced PASMC migration, whereas Jag2 knockdown decreased this migratory ability (Figure 4E). We also evaluated the effect of the Jag2/NOX2 pathway on oxidative stress levels in PASMCs. Western blot results showed that hypoxia suppressed the expression of antioxidant proteins Nrf2 and SOD2, whereas Jag2 knockdown led to their upregulation (Figure 4F). Consistent with these protein levels, Jag2 knockdown alleviated the hypoxia‐induced reduction in SOD levels, total GSH, and the GSH/oxidized glutathione ratio while decreasing hypoxia‐induced MDA levels (Figure 4G,H). Immunofluorescence results indicated that hypoxia promoted the levels of ROS compared to normoxia, but these levels were suppressed upon Jag2 knockdown (Figure 4I).

We also investigated the regulation of apoptosis in PASMCs by the Jag2/NOX2 signaling pathway. Western blot analysis showed that, under hypoxic conditions, the expression of apoptosis‐related proteins cleaved caspase‐3 and BAX in PASMCs was significantly decreased, whereas the expression of the anti‐apoptotic protein Bcl‐2 was significantly increased compared to normoxic conditions. However, the knockdown of Jag2 led to an upregulation of cleaved caspase‐3 and BAX and a downregulation of Bcl‐2 (Figure 4J). TUNEL staining (Figure 4K) and FCM (Figure 4L) confirmed that hypoxia inhibited PASMC apoptosis, whereas Jag2 knockdown promoted apoptosis.

**FIGURE 5:**
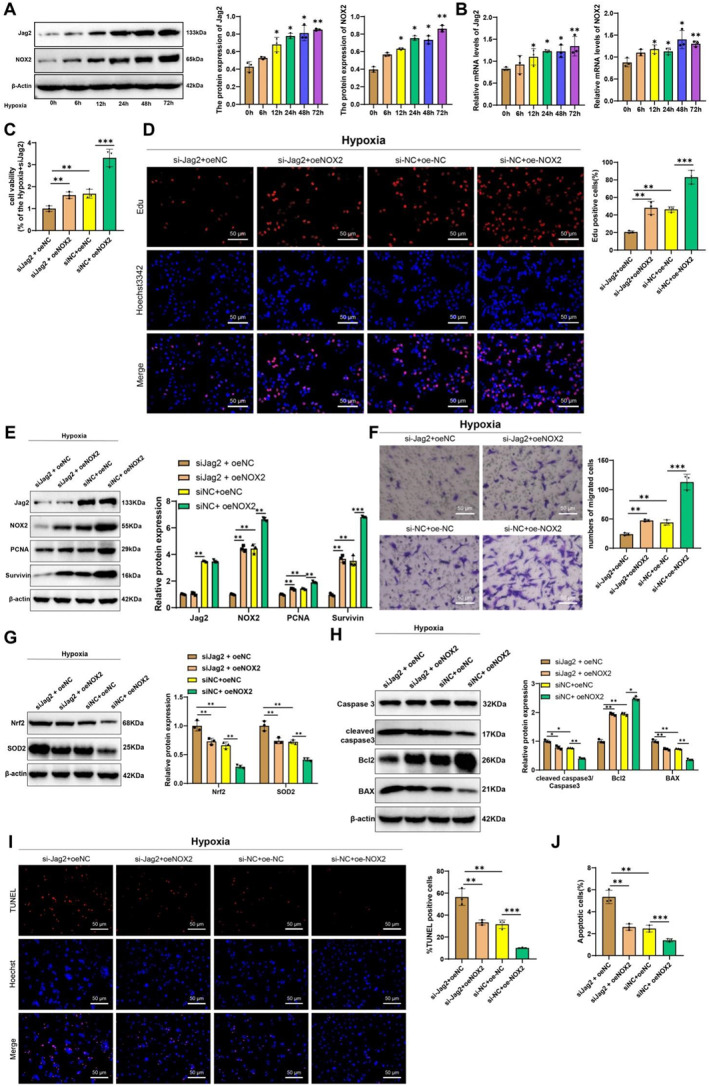
Effects of NADPH oxidase 2 (NOX2) overexpression on proliferation and migration of pulmonary artery smooth muscle cells (PASMCs) under hypoxic conditions. (A) Expression of Jag2 and NOX2 in PASMCs at various time points under hypoxic treatment detected by immunofluorescence. (B) mRNA levels of Jag2 and NOX2 in PASMCs at various time points under hypoxic treatment detected by qRT‐PCR. (C) Cell proliferation levels were measured by the CCK‐8 assay. (D) Cell proliferation levels assessed by EdU staining, scale bar = 50 μm. (E) Protein expression of Jag2, NOX2, proliferating cell nuclear antigen (PCNA), and survivin in different groups was detected by the western blot. (F) Cell migration levels were assessed using a Transwell assay. (G) Protein expression of nuclear factor erythroid 2–related factor 2 (Nrf2) and SOD2 in different groups detected by the western blot; levels of superoxide dismutase (SOD), malondialdehyde (MDA), total glutathione (GSH), reduced GSH, and oxidized GSH measured by assay kits; reactive oxygen species (ROS) levels assessed by 2',7'‐dichlorodihydrofluorescein diacetate (DCFH‐DA) staining. (H) Protein expression of caspase‐3, cleaved caspase‐3, B‐cell lymphoma 2 (Bcl2), and Bcl‐2‐associated X protein (BAX) in different groups detected by the western blot. (I) Apoptosis levels in cells of different groups were assessed by terminal deoxynucleotidyl transferase dUTP nick end labeling (TUNEL) assay, scale bar = 50 μm. (J) Apoptosis levels in cells of different groups were measured by flow cytometry (FCM). * indicates *p* < 0.05 compared to the hypoxia + siJag2 group, ** indicates *p* < 0.01 compared to the hypoxia + siJag2 group; all cell experiments were performed in triplicate.

Furthermore, after NOX2 was reexpressed in Jag2‐knockdown cells, we observed that the expression of Jag2 remained unchanged. Results from CCK‐8, EdU staining, and western blot analyses demonstrated that Jag2 knockdown significantly inhibited PASMC proliferation under hypoxia. However, reintroducing NOX2 in Jag2‐silenced cells restored the proliferation capacity. Similarly, compared with the oe‐NC group, NOX2 overexpression alone also enhanced PASMC proliferation under hypoxic conditions (Figure 5C–E). Transwell migration assays showed that Jag2 knockdown suppressed PASMC migration, whereas NOX2 re‐expression or NOX2 overexpression alone promoted cell migration under hypoxia (Figure 5F). In addition, Jag2 knockdown significantly decreased oxidative stress markers (ROS and MDA) and increased antioxidant markers (Nrf2, SOD2, and reduced GSH), whereas NOX2 reexpression reversed these effects. Overexpression of NOX2 alone also led to increased ROS and MDA levels and decreased Nrf2, SOD2, and GSH levels under hypoxia (Figure 5G). Furthermore, western blot, TUNEL staining, and FCM results showed that Jag2 knockdown promoted PASMC apoptosis, which was suppressed by either NOX2 reexpression or NOX2 overexpression (Figure 5H–J). These findings indicate that the Jag2/NOX2 pathway suppresses hypoxia‐induced apoptosis in PASMCs.

### 3.4 The Jag2/NOX2 signaling pathway mediates hypoxia‐induced vascular remodeling

**FIGURE 6:**
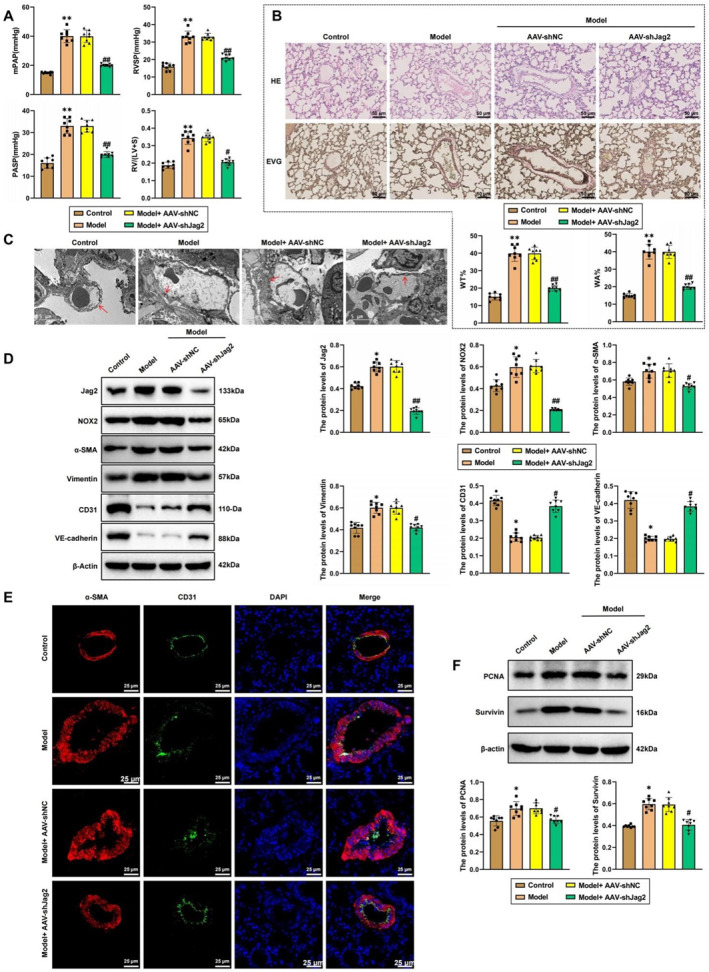
Regulation of gene expression and vascular remodeling by the Jag2/NADPH oxidase 2 (NOX2) pathway in hypoxic pulmonary arterial hypertension (PAH) rat models. (A) Measurement of mean pulmonary arterial pressure (mPAP), right ventricular systolic pressure (RVSP), pulmonary artery systolic pressure (PASP), and right ventricle (RV)/(left ventricle [LV] + S) values in each group of rats. (B) H&E and Elastica Van Gieson (EVG) staining of pulmonary artery remodeling in each group of rats (scale bar = 50 μm). (C) Transmission electron microscopy of the basal membrane morphology of pulmonary arterioles in each group of rats (scale bar = 5 μm). (D) Western blot analysis of Jag2, NOX2, alpha‐smooth muscle actin (α‐SMA), vimentin, CD31, and VE‐cadherin expression in each group. (E) Immunofluorescence detection of CD31 and α‐SMA expression in the pulmonary arteries of each group of rats (scale bar = 50 μm). (F) Western blot analysis of proliferating cell nuclear antigen (PCNA) and survivin expression in lung tissues of each group. *indicates *p* < 0.05 compared to the control group; ** indicates *p* < 0.01 compared to the control group; # indicates *p* < 0.05 compared to the model + AAV‐shNC group; ## indicates *p* < 0.01 compared to the model + AAV‐shNC group. *N* = 8.

To elucidate the role of Jag2/NOX2 signaling in hypoxia‐induced vascular remodeling in a rat model of PAH, we induced PAH using a 10% O_2_ hypoxic environment. We then measured mPAP, RVSP, PASP, and the RV/(LV + S). The results showed that, compared to the normoxia group, hypoxia significantly increased mPAP, RVSP, PASP, and RV/(LV + S), indicating the successful establishment of the hypoxic PAH rat model. In rats with Jag2 knockdown (AAV‐shJag2), these parameters were significantly decreased, suggesting that Jag2 knockdown ameliorates the hemodynamic changes associated with PAH (Figure 6A). To assess the impact of Jag2 on pathological changes in pulmonary arterioles, we performed hematoxylin and eosin (H&E) and elastic Van Gieson (EVG) staining. The results showed that, compared to the normoxia group, hypoxia increased the WA% and WT% of arterioles, indicating arterial wall thickening. In Jag2 knockdown rats, both WA% and WT% were significantly reduced (Figure 6B). Transmission electron microscopy revealed that hypoxia caused irregular thickening and visible breaks in the pulmonary artery basement membrane, which were alleviated in Jag2 knockdown rats (Figure 6C). Western blot analysis (Figure 6D) and immunofluorescence results (Figure 6E) indicated that hypoxia reduced the expression of endothelial markers CD31 and VE‐cadherin in the pulmonary artery while increasing the expression of α‐SMA and vimentin, markers of arteriole muscularization. These findings suggest that hypoxia promotes endothelial phenotype transition and vascular remodeling, whereas Jag2 knockdown attenuates these changes. Additionally, Western blot results confirmed that hypoxia significantly increased the expression of proliferation‐related proteins PCNA and survivin in pulmonary tissues, which were suppressed by Jag2 knockdown (Figure 6F). Together, these findings suggest that the Jag2/NOX2 signaling pathway contributes to hypoxia‐induced vascular remodeling in PAH.

### 3.5 Jag2/NOX2 signaling regulates inflammation, oxidative stress, and apoptosis in hypoxic PAH rats

**FIGURE 7:**
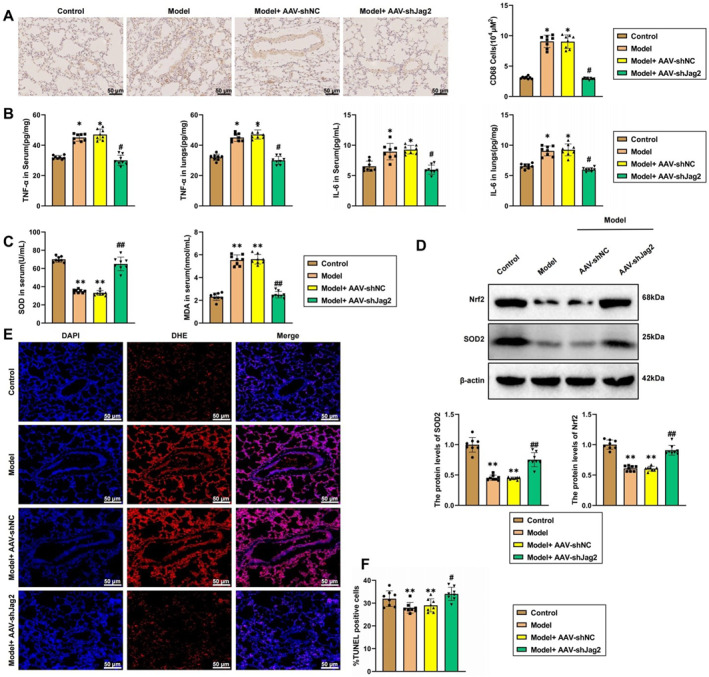
Effects of the Jag2/NADPH oxidase 2 (NOX2) pathway on inflammation, oxidative stress, and apoptosis in a hypoxic pulmonary arterial hypertension (PAH) rat model. (A) Immunohistochemistry detection of CD68 expression in the lungs of different groups of rats, scale bar = 50 μm; (B) ELISA detection of tumor necrosis factor‐alpha (TNF‐α) and IL‐6 levels in serum and lung tissue; (C) measurement of superoxide dismutase (SOD) and malondialdehyde (MDA) levels in the serum of different rat groups; (D) western blot detection of nuclear factor erythroid 2–related factor 2 (Nrf2) and SOD2 expression in lung tissue; (E) DHE staining for reactive oxygen species (ROS) levels in lung tissue, scale bar = 50 μm; (F) terminal deoxynucleotidyl transferase dUTP nick end labeling (TUNEL) detection of apoptosis levels in lung tissue, scale bar = 20 μm. *p < 0.05 compared to the control group, **p < 0.01 compared to the control group, #*p* < 0.05 compared to the model + AAV‐shNC group, ##*p* < 0.01 compared to the model + AAV‐shNC group, *N* = 8.

Inflammation, oxidative stress, and apoptosis are critical factors influencing vascular remodeling in hypoxic PAH.^33^ To determine the effect of Jag2/NOX2 signaling on inflammation, we first used immunohistochemistry to detect the number of CD68‐positive macrophages in lung tissue. The results showed that hypoxia increased the number of CD68‐positive cells around the pulmonary arteries compared to the normoxia group, whereas Jag2 knockdown significantly reduced the number of positive cells in hypoxic rats (Figure 7A). Further, we measured the levels of inflammatory cytokines released by macrophages. The results indicated that hypoxia elevated the levels of TNF‐α and IL‐6 in serum and lung tissue compared to the normoxia group, whereas Jag2 knockdown significantly reduced these proinflammatory cytokine levels (Figure 7B). These findings suggest that Jag2/NOX2 signaling promotes inflammation in hypoxic PAH rats. We also assessed oxidative stress‐related markers. Compared to the normoxia group, hypoxia reduced the expression of antioxidant factors SOD and Nrf2 while increasing MDA expression (Figure 7C). Jag2 knockdown subsequently decreased oxidative stress levels. Analysis of ROS levels revealed that hypoxia enhanced ROS levels in the pulmonary arterial endothelium and smooth muscle layers compared to the normoxia group, whereas Jag2 knockdown inhibited ROS levels (Figure 7D,E). These results indicate that Jag2/NOX2 signaling promotes oxidative stress in hypoxic PAH rats. TUNEL staining results demonstrated that hypoxia increased apoptosis levels in rat lung tissue compared to the normoxia group, whereas Jag2 knockdown promoted apoptosis levels in the pulmonary arteries (Figure 7F). Collectively, these findings suggest that Jag2/NOX2 signaling enhances inflammation and oxidative stress while inhibiting apoptosis in hypoxic PAH rats.

## 4 DISCUSSION

PAH is a fatal pulmonary vascular disease characterized by persistent PAH and vascular remodeling, which ultimately leads to right heart failure and death.^1^, ^4^, ^6^ Despite significant progress in understanding PAH, effective diagnostic and therapeutic strategies remain lacking, and the exact pathological mechanisms, especially the molecular regulatory mechanisms under hypoxic conditions, are not fully elucidated.^34^ Bioinformatics analysis is increasingly being used to identify new genes associated with PAH, potential diagnostic and prognostic biomarkers, mechanisms, and therapeutic targets.^20^, ^23^ In this study, we identified 288 DEGs from public GEO databases after the differential analysis of PAH expression profiles. KEGG and GO results revealed that pathways related to immune responses, oxidative stress, and cell death were significantly enriched in the hypoxia group. WGCNA further identified gene modules highly associated with hypoxic PAH, and RF and LASSO regression analyses identified Jag2 and Pcdh19 as key genes. Jag2, which was significantly upregulated in the hypoxia group, exhibited high diagnostic potential (AUC = 1) and was identified as a key gene in hypoxic PAH. In vitro and in vivo experiments showed that Jag2 acts as an upstream regulator of NOX2 and activates the NOX2/ROS pathway under hypoxic conditions, promoting the formation of the vascular inflammatory microenvironment, enhancing PASMC proliferation and migration, and aggravating vascular remodeling in hypoxic PAH. This study suggests that the Jag2/NOX2/ROS axis is a novel potential therapeutic target for PAH. Furthermore, Pcdh19, which was significantly downregulated in the hypoxia group, may also play a role in the pathogenesis of PAH. Pcdh19 (Protocadherin 19), a member of the nonclassical cadherin superfamily, plays important roles in neural development, cell adhesion, and signal transduction.^35^, ^36^ However, research on Pcdh19 in PAH is limited, requiring further experimental investigations.

Jag2 is a ligand in the Notch signaling pathway, which plays an essential regulatory role in cell differentiation, proliferation, and apoptosis.^37^, ^38^ Previous studies have highlighted the role of Jag2 in various diseases, including cancer and cardiovascular diseases.^39^, ^40^, ^41^ For example, Jag2 regulates tumor cell proliferation and migration via the Notch signaling pathway. However, studies on its role in PAH are relatively limited.^40^, ^42^ The NOX2/ROS signaling pathway plays a critical role in the development of many vascular diseases,^43^, ^44^ with NOX2 being a major producer of ROS. Excessive ROS leads to cellular damage, inflammation, and vascular remodeling.^45^, ^46^, ^47^ However, the specific regulatory mechanisms of the NOX2/ROS pathway in PAH remain poorly understood. This study is the first to systematically explore the role of Jag2 in hypoxic PAH, revealing that Jag2 promotes vascular inflammation and remodeling via activation of the NOX2/ROS pathway. Specifically, under hypoxic conditions, Jag2 upregulates NOX2 expression, which leads to the increased expression of proinflammatory factors and elevated oxidative stress levels, inhibits PASMC apoptosis, and promotes PASMC proliferation and migration, ultimately contributing to vascular remodeling in the PAH rat model. Previous studies have suggested that Jag2 may accelerate the development of hypoxic PAH by inhibiting the Nrf2/HO‐1 pathway and enhancing Sirtuin 1‐mediated oxidative stress, cell proliferation, and mitochondrial damage.^48^ Furthermore, Lin et al. reported that the Jagged/Notch signaling pathway exacerbates PAH progression by promoting endothelial‐to‐mesenchymal transition via GATA upregulation.^42^ Compared with previous studies, our research further clarifies the key role of Jag2 in vascular inflammation and remodeling, expanding our understanding of its molecular mechanisms in hypoxic PAH. Our study not only validates the importance of Jag2 in PAH but also provides new mechanistic insights, supporting the Jag2/NOX2/ROS pathway as a potential therapeutic target for PAH.^42^, ^48^

This study systematically reveals the critical role of Jag2 in hypoxic PAH using bioinformatics and experimental approaches. At the cellular level, we showed that Jag2 promotes PASMC proliferation and migration through NOX2. Additionally, Jag2 enhances oxidative stress and inflammation in PASMCs while inhibiting apoptosis via NOX2. In vivo, we observed that Jag2 promotes oxidative stress and inflammation in the pulmonary artery, inhibits apoptosis, and facilitates vascular remodeling in hypoxic PAH rats. These results suggest that the Jag2/NOX2/ROS pathway plays a pivotal role in the pathological progression of PAH, making Jag2 a potential therapeutic target.

Our study provides important insights into the mechanism of Jag2 in PAH, particularly its role in vascular inflammation and remodeling through the NOX2/ROS pathway. These findings offer new directions for PAH pathology research and lay the theoretical foundation for developing Jag2‐targeted therapies. Inhibiting Jag2 could effectively alleviate PAH progression and improve patient outcomes. However, the primary limitation of this study lies in the experimental models and cell lines used. The results are mainly based on rat models and in vitro cell experiments, which may not fully reflect human physiological and pathological conditions. Although our data strongly support the role of the Jag2/NOX2/ROS pathway in PAH, further studies are needed to validate its relevance across different PAH subtypes. Future research should focus on clinical validation, evaluating the safety and efficacy of Jag2 inhibitors in PAH patients and exploring other molecular mechanisms contributing to PAH pathology for comprehensive understanding and treatment.

The potential of Jag2 and NOX2 as therapeutic targets for PAH is highly promising. Inhibiting the expression of Jag2 or NOX2 could significantly alleviate the progression of PAH and improve patient outcomes. Our study provides a robust theoretical foundation for the potential application of the Jag2/NOX2/ROS pathway in PAH treatment. Future research should further validate this pathway in various animal models and clinical samples and explore its feasibility and challenges for clinical translation. Additionally, the development of specific inhibitors or targeted therapies for Jag2 and NOX2 could offer new treatment options for PAH patients.

Despite the important role of Jag2 in hypoxic PAH revealed by our study, there are several limitations. First, our research mainly focuses on rat models and in vitro cell experiments; further validation in other animal models and human clinical samples is needed to confirm its applicability. Second, although our study elucidates the critical role of the Jag2/NOX2/ROS pathway in PAH, the specific molecular regulatory mechanisms require further exploration. Future studies could employ gene editing technologies and high‐throughput screening methods to deeply analyze the regulatory mechanisms of this pathway. Furthermore, the development of specific inhibitors for Jag2 and NOX2 and their clinical application prospects need additional research and validation. In summary, our study provides new insights into the crucial role of the Jag2/NOX2/ROS pathway in PAH and outlines directions for future research and clinical applications.

## 5 CONCLUSION

This study systematically reveals the critical role of the isomerase Jag2 in hypoxic PAH using various bioinformatics and experimental approaches. At the cellular level, we demonstrated that Jag2 promotes the proliferation and migration of PASMCs via NOX2. Further analysis showed that Jag2 enhances oxidative stress and inflammation in PASMCs while inhibiting apoptosis through NOX2. By constructing a PAH rat model, we found that under hypoxic conditions, Jag2 promotes oxidative stress and inflammation in the pulmonary artery and inhibits apoptosis, thereby facilitating vascular remodeling in PAH and exacerbating hypoxic PAH (Graphical Abstract). These findings indicate that the Jag2/NOX2/ROS pathway plays a significant role in the pathological progression of PAH, making Jag2 a potential therapeutic target.

## AUTHOR CONTRIBUTIONS

Jieqing Yuan and Yunfeng Chen contributed equally to this work and share first authorship. Jieqing Yuan, Yunfeng Chen, and Siyan Wu performed the experiments and analyzed the data. Hai Shi and Yuan Dong were responsible for the bioinformatics analysis and assisted with data interpretation. Yu Han contributed to the animal model establishment and histological analysis. Wenjie Cui designed and supervised the study, provided critical revisions, and finalized the manuscript. All authors read and approved the final version of the manuscript.

## CONFLICT OF INTEREST STATEMENT

The authors declare no conflicts of interest.

## ETHICS STATEMENT

All animal experiments were conducted in compliance with institutional and national guidelines for the care and use of laboratory animals. The study protocol was approved by the Animal Ethics Committee of the Affiliated Xuzhou Municipal Hospital of Xuzhou Medical University (Approval No. 20231112). No human participants, human data, or human tissue were involved in this study.

## CONSENT

Not applicable, as this manuscript does not include data from any individual person.

## Supporting information

Supporting Information S1

Figure S1

Figure S2

Table S1

Table S2

## Data Availability

The datasets used and analyzed in the current study are available from the corresponding author on reasonable request.
