# Supplementary material for: Unveiling the role of Jagged2 in hypoxic pulmonary arterial hypertension: A NOX2‐mediated pathway
Source: J Cell Commun Signal. 2025 Nov 19;19(4):e70032. doi: 10.1002/ccs3.70032 (PMC12629663; doi:10.1002/ccs3.70032)
Supplement: Supplementary file 1 — Supporting Information S1 [file CCS3-19-e70032-s004.docx]

**Figure S1. Integration of GSE72181 and GSE186996 Datasets.**

Note: (A) Box plot of GEO data distribution before batch effect removal; (B) Box plot of GEO data distribution after batch effect removal; (C) Intersection of genes in the GSE72181 and GSE186996 datasets.

**Figure S2. Identification of PASMCs and Efficiency Assessment of Jag2 Knockdown.**

Note: (A) Immunofluorescence staining of α-SMA for PASMC identification, scale bar = 25 μm ; (B) Efficiency of Jag2 siRNA knockdown evaluated by Western blot and RT-PCR. ** indicates *p* < 0.01 compared to the siNC group. All cellular experiments were repeated three times.
