# Supplementary figures and images for: Unveiling the role of Jagged2 in hypoxic pulmonary arterial hypertension: A NOX2‐mediated pathway
Source: J Cell Commun Signal. 2025 Nov 19;19(4):e70032. doi: 10.1002/ccs3.70032 (PMC12629663; doi:10.1002/ccs3.70032)

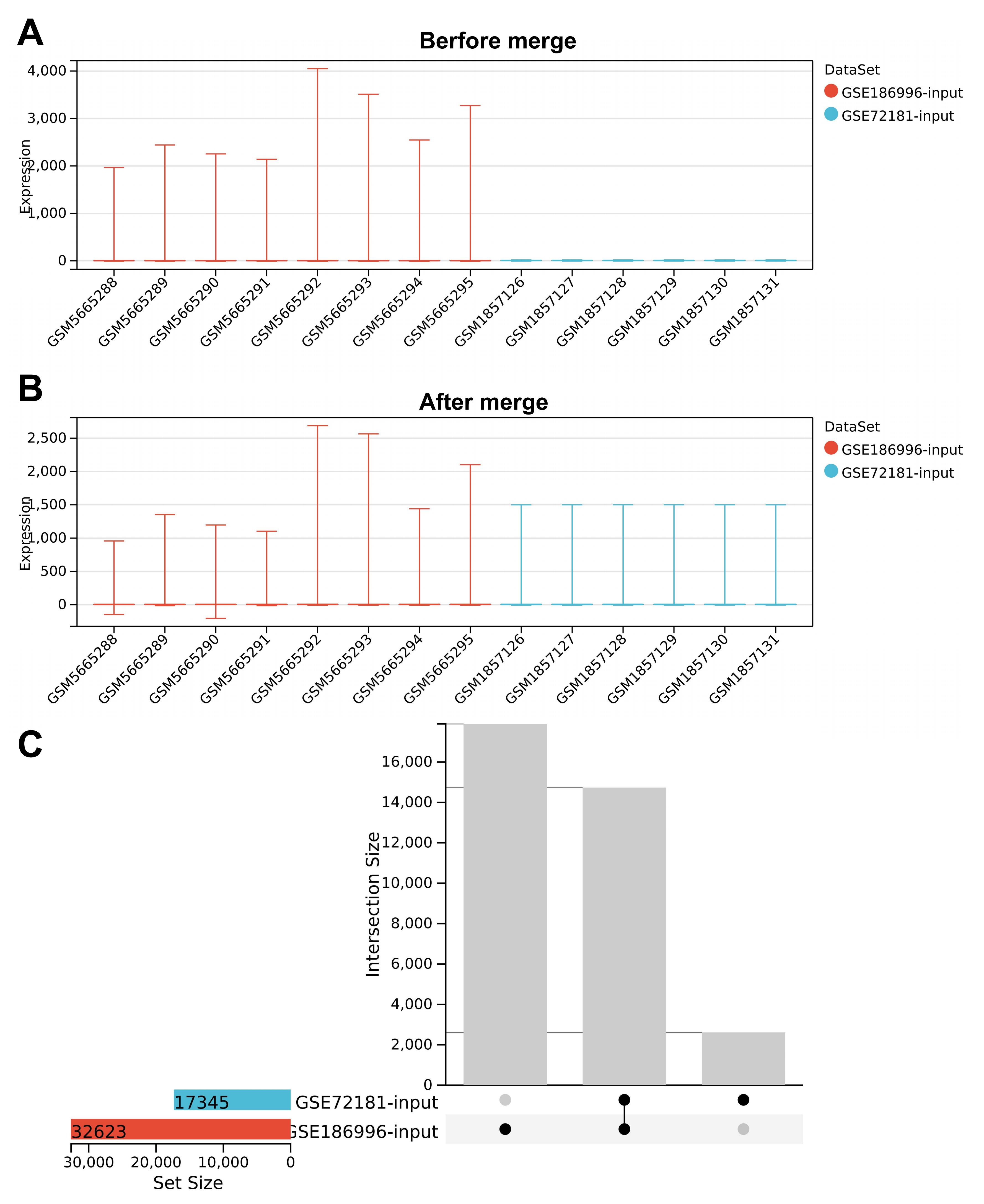

Supplement: Supplementary file 2 — Figure S1 [file CCS3-19-e70032-s002.jpg]

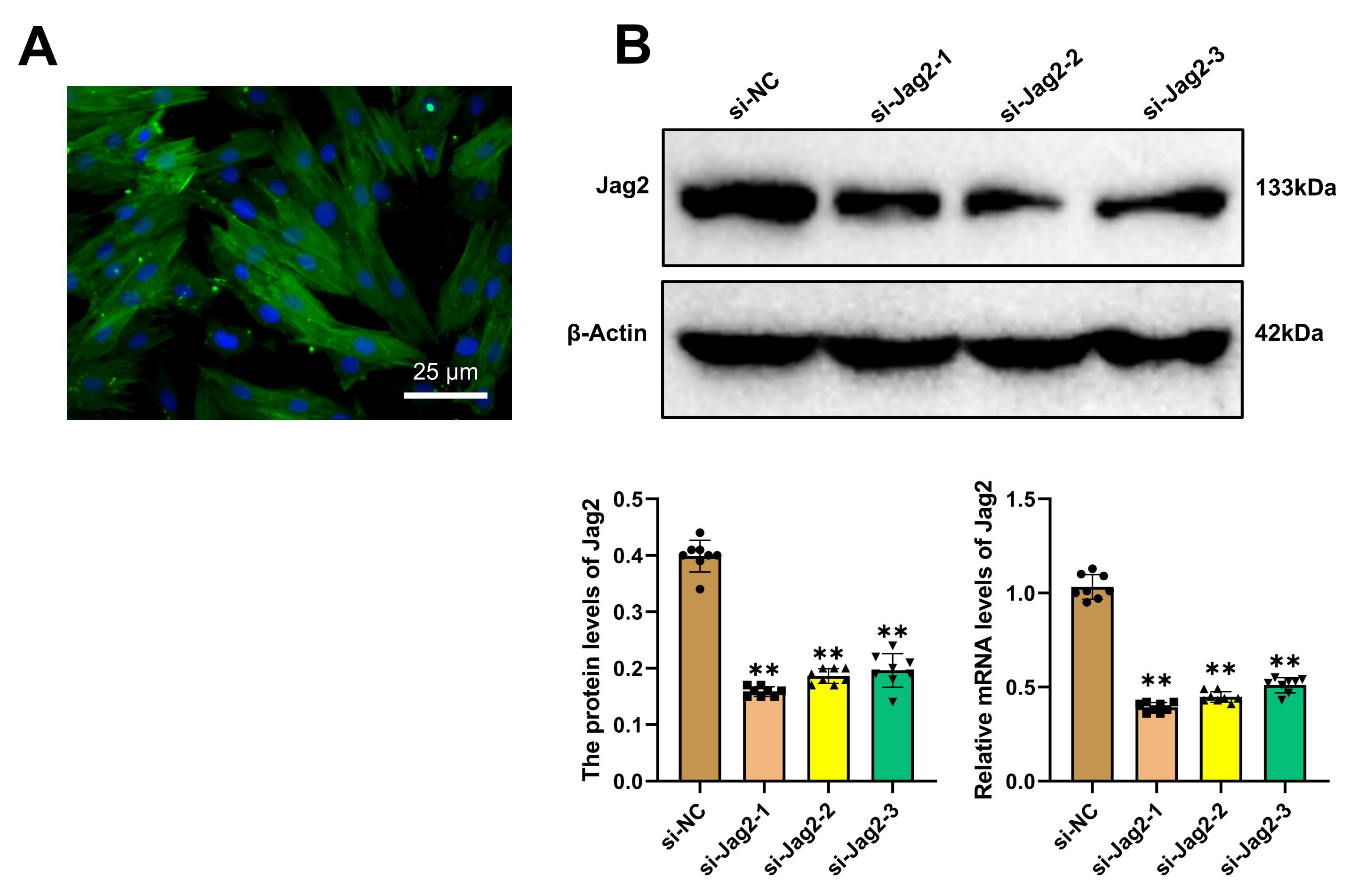

Supplement: Supplementary file 3 — Figure S2 [file CCS3-19-e70032-s001.jpg]
