# Supplementary material for: Unveiling the role of Jagged2 in hypoxic pulmonary arterial hypertension: A NOX2‐mediated pathway
Source: J Cell Commun Signal. 2025 Nov 19;19(4):e70032. doi: 10.1002/ccs3.70032 (PMC12629663; doi:10.1002/ccs3.70032)
Supplement: Supplementary file 4 — Table S1 [file CCS3-19-e70032-s003.docx]

**Table S1. Basic information of GEO datasets.**

| **Dataset** | **Platform** | **Number of samples (normoxia/hypoxia)** | **Organism** | **Tissue type** |
| --- | --- | --- | --- | --- |
| GSE72181 | GPL7294 | 3:3 | Rattus norvegicus | Lung tissue |
| GSE186996 | GPL20084 | 4:4 | Rattus norvegicus | Lung tissue |
