# Supplementary material for: Unveiling the role of Jagged2 in hypoxic pulmonary arterial hypertension: A NOX2‐mediated pathway
Source: J Cell Commun Signal. 2025 Nov 19;19(4):e70032. doi: 10.1002/ccs3.70032 (PMC12629663; doi:10.1002/ccs3.70032)
Supplement: Supplementary file 5 — Table S2 [file CCS3-19-e70032-s005.docx]

**Table S2. RT-qPCR primer sequences.**

| **Gene** | **Primer Sequence (5'- 3')** |
| --- | --- |
| Jag2(Rat) | F: 5' - AGCTGCCTTATTTTTAGGCGA -3' |
|  | R: 5' - ACCTTGGCCTGGTACTCCTTA -3' |
| NOX2(Rat) | F: 5' -TGCAGCCAAATGCCCTGATA-3' |
|  | R: 5' - AAGGCGCTCTGAAACCATGA-3' |
| β-actin(Rat) | F: 5' - CCCGCGAGTACAACCTTCTT -3' |
|  | R: 5' - CGCAGCGATATCGTCATCCA -3' |
